# Omni-directional and broadband acoustic anti-reflection and universal acoustic impedance matching

**DOI:** 10.1515/nanoph-2021-0650

**Published:** 2022-02-25

**Authors:** Ku Im, Q-Han Park

**Affiliations:** Department of Physics, Korea University, Seoul, 02841, Korea

**Keywords:** acoustic wave, anti-reflection, impedance matching, metamaterial, spatio-temporal dispersion

## Abstract

The mechanism of the perfect anti-reflection of acoustic waves, regardless of frequency and incident angle, is presented. We show that reflections at a planar interface between two different acoustic media can be removed by adding a nonlocal metamaterial that compensates for the impedance mismatch. The properties required of a nonlocal metamaterial are explicitly specified through spatio-temporally dispersive mass density and bulk modulus. We analyze the characteristics of spatio-temporal dispersion according to the thickness of the matching layer. We discuss the issue of the total internal reflection caused by conventional matching layers and explain how our nonlocal matching layer avoids this. The practical design of our nonlocal layer using metamaterials is explained. The omni-directional frequency-independent behavior of the proposed anti-reflection matching layer is confirmed through explicit numerical calculation using the finite element method, and comparisons made to the conventional quarter-wave matching layer approach.

## 1 Introduction

Acoustic waves reflect at the interface between two different media, due to the discontinuity of acoustic impedance. This reflection is often undesirable as it can damage wave generators or medium materials through the formation of constructive interference inside media [1], [2], [3], [4]. In addition, acoustic reflection is a hindrance for industrial applications such as sonography, acoustic power transfer, and perfect acoustic absorption, for which the maximum transmission of acoustic energy is preferred [5], [6], [7], [8], [9], [10]. Various efforts have been made to remove unwanted reflection by applying a matching layer made of porous materials [7], single- or multi-layered materials [11, 12], graded index materials [13], [14], [15], and acoustic metamaterials [3, 16, 17]. Although these matching layers compensate for the impedance mismatch, their performance has previously been restricted to a specific frequency or incident angle.

The Mason’s equivalent circuit model [18] and the Krimholtz, Leedom, and Matthaei transmission line method [19] have been utilized to develop anti-reflection matching layers for broader frequency band operations [20]. However, this bandwidth broadening was achieved at the cost of unnecessary acoustic wave absorption inside the matching layer, which reduced the efficiency of energy transfer [4]. Moreover, since the circuit model does not consider the incident angle, it does not help in the design of acoustic impedance matching layers for varied incidence angles, and this limits its application to the non-destructive wide-angle inspection of industrial materials [21]. In short, so far, no systematic approach has been devised for removing the impedance mismatch between two different acoustic media regardless of incident angle and frequency.

Here, we derive the material condition for universal acoustic impedance matching (UAIM) from acoustic wave equations, allowing us to remove unwanted reflection regardless of frequencies and incident angles. Inspired by the electromagnetic universal impedance matching theory [22], we first reformulate the acoustic wave equation in terms of impedance function and then express the material parameters explicitly as functions of the impedance. We show that, in order to obtain omni-directional and broadband acoustic anti-reflection, the impedance of a matching layer should vary with respect to incident angles and frequencies, thereby resulting in a matching layer featuring spatially and temporally dispersive material parameters. For a specific UAIM example, we present a homogeneous UAIM layer and compare its anti-reflective performance to a conventional quarter-wave anti-reflection (QAR) matching layer. We also address the issue of the total internal reflection that could be caused by conventional matching layers and explain how our UAIM matching layer avoids it. We also demonstrate the perfect anti-reflective performance of the UAIM layer both analytically and numerically.

## 2 Theory of UAIM

### 2.1 Acoustic wave equation in terms of impedance function

Acoustic waves are a special case of elastic medium waves that are characterized according to the deformation and stress fields obeying the generalized Hooke’s law and Newton’s second law [23]. An isotropic linear elastic medium characterized by the bulk modulus and shear modulus supports both compressional and shear waves. When the bulk modulus far outweighs the shear modulus, the shear wave is negligible, and the dominant compressional wave is known as the acoustic wave. In this acoustic approximation, the stress and deformation fields are described by the acoustic pressure field p and the deformation velocity field $\vec{u}$ and the wave equation becomes the acoustic wave equation:

(2.1.1) $$\partial_{t}p+K\nabla \cdot \vec{u}=0,$$

(2.1.2) $$\nabla p+\rho \partial_{t}\vec{u}=0.$$

where ρ is the mass density of the medium, K is the bulk modulus, and $\partial_{t}$ refers to the time-derivative.

**Figure 1:**
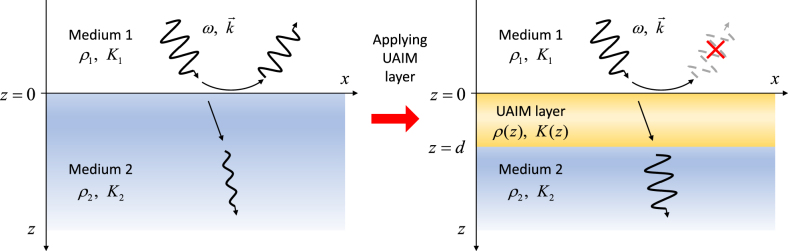
UAIM layer enabling the complete removal of acoustic reflection regardless of frequencies and incident angles.

As shown in Figure 1, we consider a linear and time-independent medium possessing rotational symmetry about the z -axis and translational symmetry in the xy -plane. We first restrict acoustic waves to time-harmonic fields s.t.

(2.1.3) $$\begin{array}{l} p=P\left(z\right)\exp\left(ik_{x}x+ik_{y}y-i\omega t\right),\\ \vec{u}=\vec{V}\left(z\right)\exp\left(ik_{x}x+ik_{y}y-i\omega t\right), \end{array}$$

where P(z) and $\vec{V}\left(z\right)$ are the z -dependent part of p and $\vec{u}$ respectively, ω is the angular frequency and $k\equiv \sqrt{k_{x}^{2}+k_{y}^{2}}$ is the magnitude of the transverse wave vector ${\vec{k}}^{∥}=k_{x}\hat{x}+k_{y}\hat{y}$ . Substituting these time-harmonic fields in Eqs. (2.1.1) and (2.1.2), we find the second order differential equation in self-adjoint form for the pressure field

(2.1.4) $$\rho \left(z\right)\;\partial_{z}\left(\frac{1}{\rho \left(z\right)}\partial_{z}P\left(z\right)\right)+\left(\frac{\omega^{2}\rho \left(z\right)}{K\left(z\right)}-k^{2}\right)P\left(z\right)=0.$$

The velocity components are directly obtained from pressure field by

(2.1.5) $$V_{x}=\frac{k_{x}}{\omega \rho \left(z\right)}P\left(z\right),\;\;V_{y}=\frac{k_{y}}{\omega \rho \left(z\right)}P\left(z\right),\;V_{z}=\frac{\partial_{z}P\left(z\right)}{i\omega \rho \left(z\right)}=\frac{P\left(z\right)}{Z\left(z\right)}\;,\;\;\;\;\left(\;Z\left(z\right)\equiv \frac{i\omega \rho \left(z\right)}{\partial_{z}\;\ln\;P\left(z\right)}\right).$$

where in the last step, we introduced impedance Z . In the case of a homogeneous medium with constant ρ and K , we find $P=\exp\left(ik_{z}z\right)$ for $k_{z}\equiv \sqrt{\omega^{2}\rho /K-k^{2}}$ and impedance $Z=\omega \rho /k_{z}$ , which is constant. For z-dependent ρ and K , the impedance function Z(z) , as the ratio of p(z) to $u_{z}\left(z\right)$ , is particularly useful in our dimensionally reduced acoustic Eq. (2.1.4). One can readily check that Eq. (2.1.4) is equivalent to

(2.1.6) $$i\omega \rho =\partial_{z}Z+\frac{i\omega }{K}\left(1-\frac{k^{2}}{\omega^{2}\rho /K}\right)Z^{2}.$$

If the medium is lossless with real parameters ρ and K , we can express the real and imaginary part of the impedance function by $Z\left(z\right)=Z_{\text{R}}\left(z\right)+iZ_{\text{I}}\left(z\right)$ and the real and imaginary parts of Eq. (2.1.6) separately by

(2.1.7) $$\rho =\frac{1}{\omega }\left[\partial_{z}Z_{\text{I}}+\left(Z_{\text{R}}^{2}-Z_{\text{I}}^{2}\right)\frac{\partial_{z}Z_{\text{R}}}{2Z_{\text{R}}Z_{\text{I}}}\right],$$

(2.1.8) $$K={\left[\frac{\partial_{z}Z_{\text{R}}}{2\omega Z_{\text{R}}Z_{\text{I}}}+\frac{k^{2}}{\omega^{2}\rho }\right]}^{-1}.$$

This result is significant. We have achieved a direct correspondence between the material parameters ρ , K and the field variables expressed in terms of the impedance function Z(z) . Note that the impedance function can be any arbitrary function as long as it does not cause singularities of ρ and K . In physical situations, we may need to apply specific boundary conditions to the impedance function, but otherwise Z(z) is arbitrary. Importantly, the impedance function satisfying the required boundary conditions provides a direct inverse scattering scheme through Eqs. (2.1.7) and (2.1.8) for material parameter reconstruction.

### 2.2 Reflection-zero conditions and a constant UAIM layer

A prominent application of the direct inverse scattering scheme is the perfect anti-reflection of an acoustic wave. We seek for a universal acoustic impedance matching (UAIM) layer, which can remove reflection regardless of wave frequency and incidence angle. Consider an acoustic wave propagating through the planar interface between two different homogeneous media of mass densities $\rho_{1}$ , $\rho_{2}$ and bulk moduli $K_{1}$ , $K_{2}$ respectively, as shown in Figure 1. A matching layer of thickness *d*, mass density ρ and bulk modulus K is positioned at the interface. From Eq. (2.1.4), we know that if P is the solution $P^{*}$ is also the solution for the real ρ and K . Inside both medium 1 and medium 2, we find $P=\exp\left(ik_{z}z\right)$ so that P and $P^{*}$ represent the right- and left-propagating modes, respectively. If a wave is incident with angular frequency ω and transverse wavenumber k , the wave field components in each region, normalized by the amplitude of the incident field, can be written as

(2.2.1) $$\begin{array}{c} \begin{array}{cccccc} P\;\left(z<0\right) & =e^{ik_{1,z}z}+re^{-ik_{1,z}z}, & P\left(0<z<d\right) & =AP_{\text{M}}\left(z\right)+BP_{\text{M}}^{*}\left(z\right), & P\left(d<z\right) & =te^{ik_{2,z}\left(z-d\right)},\\ V_{z}\left(z<0\right) & =\frac{1}{Z_{1}}e^{ik_{1,z}z}-r\frac{1}{Z_{1}}e^{-ik_{1,z}z}, & V_{z}\left(0<z<d\right) & =A\frac{P_{\text{M}}\left(z\right)}{Z_{\text{M}}\left(z\right)}-B\frac{P_{\text{M}}^{*}\left(z\right)}{Z_{\text{M}}^{*}\left(z\right)}, & V_{z}\left(d<z\right) & =\frac{1}{Z_{2}}te^{ik_{2,z}\left(z-d\right)}, \end{array}\\ k_{j,z}=\sqrt{\frac{\omega^{2}\rho_{j}}{K_{j}}-k^{2}}=\frac{\omega }{c_{j}}\cos\;\theta_{j},\;Z_{j}=\frac{Z_{j}^{\left(0\right)}}{\cos\;\theta_{j}},\;Z_{j}^{\left(0\right)}\equiv \sqrt{\rho_{j}K_{j}},\;\cos\;\theta_{j}\equiv \sqrt{1-\frac{c_{j}^{2}k^{2}}{\omega^{2}}},\;c_{j}\equiv \sqrt{K_{j}/\rho_{j}}, \end{array}$$

where r and t are the reflection and transmission coefficients, A and B are the coefficients for the normalized pressure modes $P_{\text{M}}$ , $P_{\text{M}}^{*}$ of the matching layer, and $Z_{\text{M}}$ is the corresponding impedance function. Here, $Z_{j}^{\left(0\right)}$ , $\cos\;\theta_{j}$ , and $c_{j}$ are the specific acoustic impedance (SAI), directional cosine, and speed of the propagating waves in the j -th medium, respectively. Coefficients including r and t are determined by requiring the continuity condition of the pressure field p and the velocity field component $u_{z}$ . Applying the continuity of p and $u_{z}$ at each interface, we may present the boundary condition in a matrix form,

(2.2.2) $$\left(\begin{array}{cc} 1 & 1\\ \frac{1}{Z_{1}} & -\frac{1}{Z_{1}} \end{array}\right)\left(\begin{array}{c} 1\\ r \end{array}\right)=\left(\begin{array}{cc} P_{\text{M}}\left(0\right) & P_{\text{M}}^{*}\left(0\right)\\ \frac{P_{\text{M}}\left(0\right)}{Z_{\text{M}}\left(0\right)} & -\frac{P_{\text{M}}^{*}\left(0\right)}{Z_{\text{M}}^{*}\left(0\right)} \end{array}\right)\left(\begin{array}{c} A\\ B \end{array}\right),$$

(2.2.3) $$\left(\begin{array}{cc} P_{\text{M}}\left(d\right) & P_{\text{M}}^{*}\left(d\right)\\ \frac{P_{\text{M}}\left(d\right)}{Z_{\text{M}}\left(d\right)} & -\frac{P_{\text{M}}^{*}\left(d\right)}{Z_{\text{M}}^{*}\left(d\right)} \end{array}\right)\left(\begin{array}{c} A\\ B \end{array}\right)=\left(\begin{array}{cc} 1 & 1\\ \frac{1}{Z_{2}} & -\frac{1}{Z_{2}} \end{array}\right)\left(\begin{array}{c} t\\ 0 \end{array}\right).$$

Eliminating A and B , we find

(2.2.4) $$\begin{array}{c} r=\frac{V_{\text{M}}\left(0\right)V_{\text{M}}^{*}\left(d\right)\left[Z_{\text{M}}\left(0\right)-Z_{1}\right]\left[Z_{2}+Z_{\text{M}}^{*}\left(d\right)\right]+V_{\text{M}}^{*}\left(0\right)V_{\text{M}}\left(d\right)\left[Z_{\text{M}}^{*}\left(0\right)+Z_{1}\right]\left[Z_{2}-Z_{\text{M}}\left(d\right)\right]}{V_{\text{M}}\left(0\right)V_{\text{M}}^{*}\left(d\right)\left[Z_{\text{M}}\left(0\right)+Z_{1}\right]\left[Z_{2}+Z_{\text{M}}^{*}\left(d\right)\right]+V_{\text{M}}^{*}\left(0\right)V_{\text{M}}\left(d\right)\left[Z_{\text{M}}^{*}\left(0\right)-Z_{1}\right]\left[Z_{2}-Z_{\text{M}}\left(d\right)\right]},\\ t=\frac{2V_{\text{M}}\left(d\right)V_{\text{M}}^{*}\left(d\right)Z_{2}\left[Z_{\text{M}}\left(d\right)+Z_{\text{M}}^{*}\left(d\right)\right]}{V_{\text{M}}\left(0\right)V_{\text{M}}^{*}\left(d\right)\left[Z_{\text{M}}\left(0\right)+Z_{1}\right]\left[Z_{2}+Z_{\text{M}}^{*}\left(d\right)\right]+V_{\text{M}}^{*}\left(0\right)V_{\text{M}}\left(d\right)\left[Z_{\text{M}}^{*}\left(0\right)-Z_{1}\right]\left[Z_{2}-Z_{\text{M}}\left(d\right)\right]}, \end{array}$$

where $V_{\text{M}}\left(z\right)\equiv P_{\text{M}}\left(z\right)/Z_{\text{M}}\left(z\right)$ is the velocity mode function inside the matching layer. If r is zero for all ω and k , the acoustic impedance is matched universally. This can be simply achieved by imposing the boundary condition:

(2.2.5) $$Z_{\text{M}}\left(0\right)=Z_{1},\;Z_{\text{M}}\left(d\right)=Z_{2}.$$

For any $Z_{\text{M}}$ satisfying (2.2.5), Eqs. (2.1.7) and (2.1.8) present the material parameters required for UAIM for perfect anti-reflection.

One simple case of UAIM is when the material parameters ρ and K are both constant. Assuming ρ and K are constant, we may rewrite Eq. (2.1.6) as

(2.2.6) $$\partial_{z}Z_{\text{M}}=\frac{i}{\eta }\left(\beta^{2}-\eta^{2}Z_{\text{M}}^{2}\right)\text{for}\;\;\;\;\eta \equiv \frac{\omega }{K}\left(1-\frac{k^{2}}{\omega^{2}\rho /K}\right),\;\beta \equiv \sqrt{\omega \rho \eta }$$

with the newly defined constants η,β . Since η/ω accounts for the compressibility and β represents the propagation wavenumber, we assume constants η and β to be positive without loss of generality. Then, Eq. (2.2.6) can be integrated to yield

(2.2.7) $$Z_{\text{M}}\left(z\right)=\frac{Z_{\text{M}}\left(0\right)\cos\;\beta z+i\frac{\beta }{\eta }\sin\;\beta z}{\cos\;\beta z+i\frac{\eta }{\beta }Z_{\text{M}}\left(0\right)\sin\;\beta z}.\;$$

Boundary condition (2.2.5) can be satisfied by choosing $\beta =\frac{2n+1}{2d}\pi$ and $\eta =\frac{\beta }{\sqrt{Z_{1}Z_{2}}}$ with a positive integer n . This leads to

(2.2.8) $$\rho =\frac{\kappa_{n}}{\omega }〈Z〉,\;K=\frac{\omega \kappa_{n}}{\kappa_{n}^{2}+k^{2}}〈Z〉,\;\kappa_{n}\equiv \frac{2n+1}{2d}\pi ,\;〈Z〉\equiv \sqrt{Z_{1}Z_{2}},$$

where 〈Z〉 is the geometric mean of the impedance of media 1 and 2. Here, the mass density and the bulk modulus should depend on ω and k in order for the anti-reflection layer to cover all frequencies and incident angles. If the transverse momentum k is zero, the fundamental mode ( n=0 ) in Eq. (2.2.8) describes the well-known quarter-wave anti-reflection (QAR) condition. Thus, the matching layer with constant material parameters in (2.2.8) is an omnidirectional generalization of the QAR layer.

## 3 Results and discussion

### 3.1 Spatial and temporal dispersions of the constant UAIM layer

**Figure 2:**
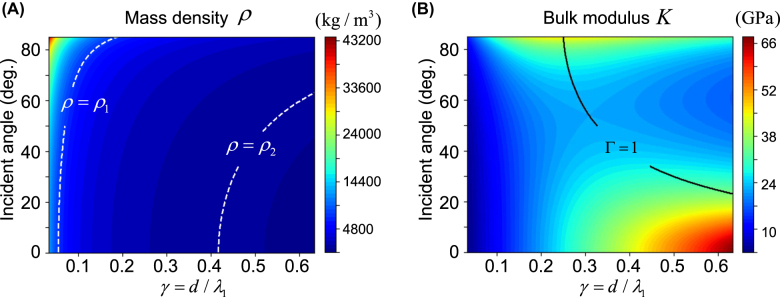
Material dispersions (A) Mass density ρ and (B) bulk modulus κ for the constant UAIM layer with respect to *θ*_1_ and the ratio *γ*. Here, *ρ*_1_ and *ρ*_2_ are the mass densities of the PZT4 ceramic and water, respectively.

The UAIM layer with constant material parameters in (2.2.8) exhibits both spatial and temporal dispersion. To understand the dispersive behavior, we consider the example of a constant UAIM layer between the PZT4 ceramic ( $\rho_{1}=7500\;\text{kg}/{\text{m}}^{-3}$ , $K_{1}=120\;\text{GPa}$ ), which is a typical material for the ultrasound probe, and water ( $\rho_{2}=998\;\text{kg}/{\text{m}}^{-3}$ , $K_{2}=2.20\;\text{GPa}$ ). These two materials cause a huge impedance mismatch and the resulting high reflection reaches 82 percent. As the impedance $Z_{1}$ and $Z_{2}$ of the incident and transmitted waves vary with respect to the incident angle $\theta_{1}$ and the frequency f=ω/2π , the matching layer, with material parameters depending on $Z_{1}$ and $Z_{2}$ , is also both spatially and temporally dispersive. Figure 2 presents the contour plots of the material parameters ρ and K constituting the constant UAIM layer of thickness d . They are plotted varying incidence angle $\theta_{1}$ and the wavelength normalized thickness $\gamma \equiv d/\lambda_{1}$ where $\lambda_{1}=c_{1}/f$ is the wavelength inside medium 1. To better understand the dispersion behavior, we consider only the fundamental mode ( n=0 ) in Eq. (2.2.8) and introduce a dimensionless parameter $\Gamma \equiv 4\gamma \;\sin\;\theta_{1}$ , which measures the relative degree of grazing incidence ( Γ=0 for normal incidence).

If Γ≪1 , $\kappa_{0}$ is dominant over k and the bulk modulus K of the UAIM layer becomes approximately proportional to γ , whereas its mass density is inversely proportional to γ (without approximation),

(3.1.1) $$\rho =\frac{1}{4\gamma }\sqrt{\frac{\rho_{1}}{K_{1}}}\frac{{\left[\rho_{1}\rho_{2}K_{1}K_{2}\right]}^{1/4}}{{\left[1-\sin^{2}\theta_{1}\right]}^{1/4}{\left[1-\sin^{2}\theta_{2}\right]}^{1/4}},\;\;\;\;\;\;\;\;\sin^{2}\theta_{2}=\frac{\rho_{1}/K_{1}}{\rho_{2}/K_{2}}\sin^{2}\;\theta_{1}$$

(3.1.2) $$K\approx 4\gamma \sqrt{\frac{K_{1}}{\rho_{1}}}\frac{{\left[\rho_{1}\rho_{2}K_{1}K_{2}\right]}^{1/4}}{{\left[1-\sin^{2}\theta_{1}\right]}^{1/4}{\left[1-\sin^{2}\theta_{2}\right]}^{1/4}}$$

**Figure 3:**
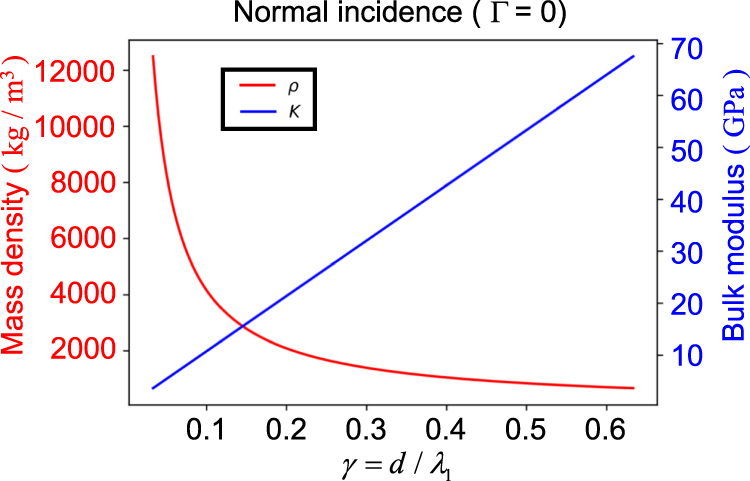
Temporal dispersion for the normal incidence.

The temporal dispersion of the normal incidence case ( Γ=0 ) is shown in terms of wavelength normalized thickness γ in Figure 3. For fixed d , γ is proportional to frequency. To obtain a parameter set for the matching layer, we may alternatively fix wavelength $\lambda_{1}$ and vary thickness d . The freedom to choose any thickness *d*, e.g., a thicker matching layer of smaller mass density, could reduce burdens related to material choice and manufacturing.

**Figure 4:**
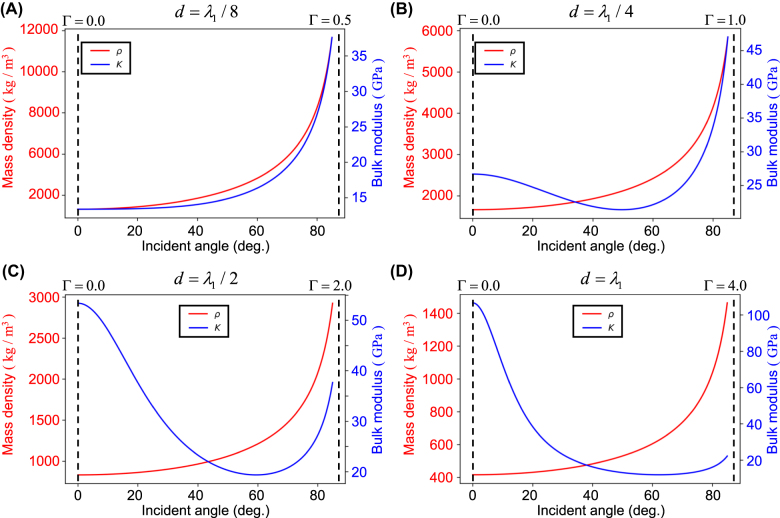
Spatial dispersion depending on the thickness to wavelength ratio of (A) *γ* = 1/8, (B) *γ* = 1/4, (C) *γ* = 1/2, and (D) *γ* = 1.

To focus on the spatial dispersion behavior, we fix wavelength $\lambda_{1}$ for different cases of d and vary the incidence angle, as shown in Figure 4. For small Γ≪1 (Figure 4A), ρ and K show similar spatial dispersion behaviors. Both the mass density and the bulk modulus are inversely proportional to the geometric mean of the cosines, $\sqrt{\cos\;\theta_{1}\;\cos\;\theta_{2}}$ , and they both show increasing behavior as the incidence angle increases. As Γ increases, spatial dispersion gets more involved as shown in Figure 4B–D. Overall, spatial dispersion becomes more pronounced as the thickness grows.

So far, we have described the properties of the spatio-temporal dispersion of a UAIM layer without specific design rules. Here, we only point out that metamaterials are intrinsically nonlocal materials. Recently, Liu et al. proposed a non-local acoustic metamaterial [24] possessing unusual spatio-temporal dispersion inspired by the shifted spatial dispersion occurring in electromagnetic media [25]. By adjusting the parameters of the unit cell metamaterial structure, the equal frequency contours of a crystal band structure can be manipulated so as to achieve the required dispersions for the constant UAIM layer.

### 3.2 Total internal reflection

**Figure 5:**
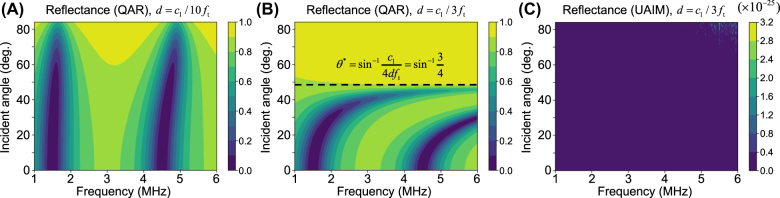
Reflection contour plot of (A) QAR of *d* = *c*_1_/10*f*_t_, (B) QAR of *d* = *c*_1_/3*f*_t_, and (C) constant UAIM of *d* = *c*_1_/3*f*_t_, where *f*_t_ = 1.5 MHz is the target frequency of the QAR.

**Figure 6:**
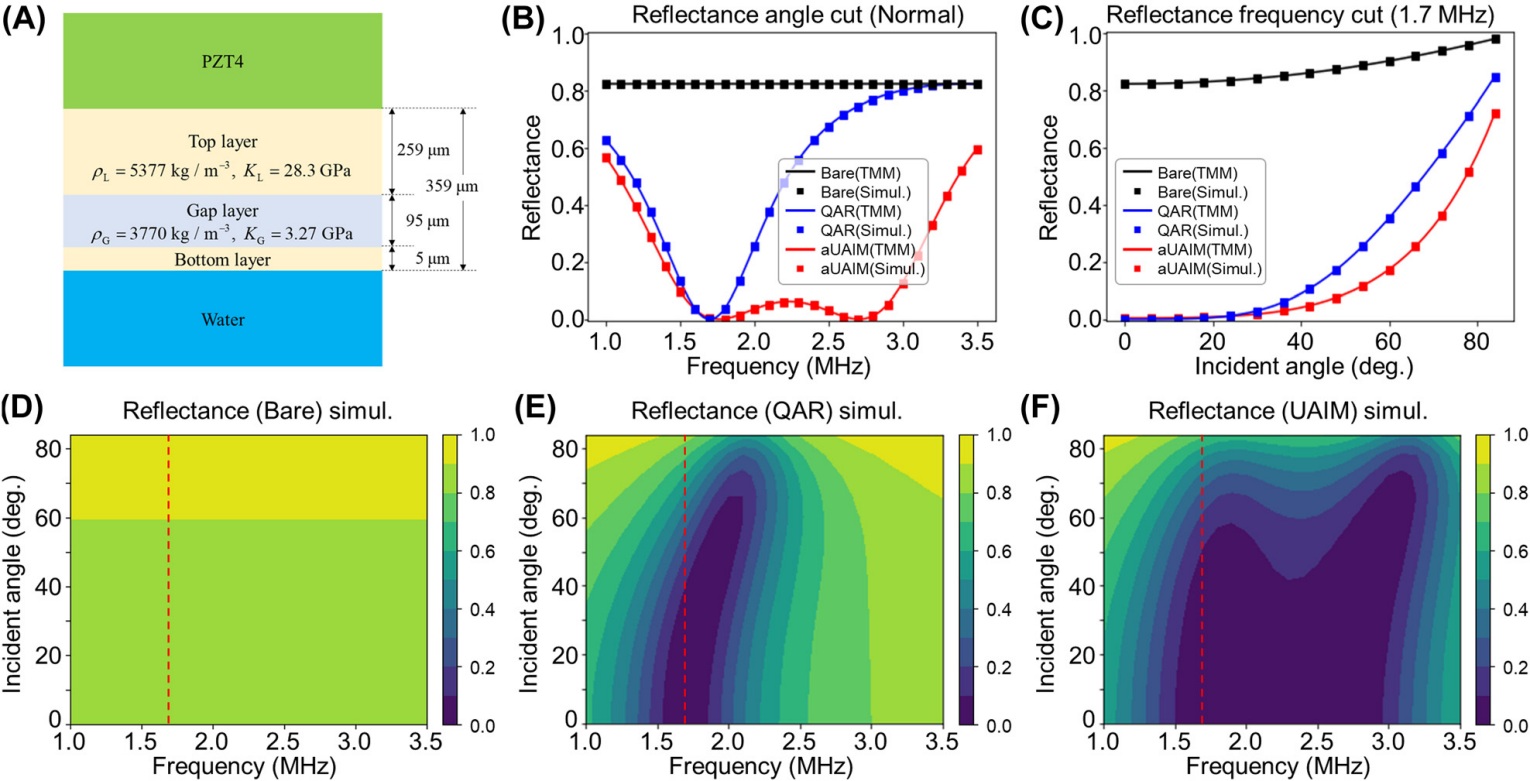
Reflection spectra of sound wave for varying incident angles with and without matching layers via COMSOL simulation. (A) Schematic design of the aUAIM layer. (B) Reflection spectra for normal incidence. (C) Angle dependence of the reflectance at frequency *f* = 1.7 MHz. Solid lines in (B) and (C) refer to the analytic solutions for each case, and squares represent the simulation results (black: bare substrate, blue: QAR, red: aUAIM). (D)–(F) Contour plots of reflectance versus incident angle and frequency for the substrate without matching layer (D), with the ideal QAR layer (E) and with the aUAIM layer (F).

The thickness d of the matching layer should be carefully chosen considering total internal reflection. Without a matching layer, total internal reflection does not occur when $c_{2}<c_{1}$ . However, adding a matching layer could result in total internal reflection even for $c_{2}<c_{1}$ , as demonstrated in Figure 5. For instance, consider a conventional QAR designed with the mass density and the bulk modulus

(3.2.1) $$\rho_{\text{Q}}=\frac{1}{4df_{\text{t}}}\sqrt{Z_{1}^{\left(0\right)}Z_{2}^{\left(0\right)}},\;K_{\text{Q}}=4df_{\text{t}}\sqrt{Z_{1}^{\left(0\right)}Z_{2}^{\left(0\right)}},$$

where $f_{\text{t}}$ is the target frequency of the QAR. The SAI $Z_{\text{Q}}^{\left(0\right)}$ of the matching layer is constant ( $Z_{\text{Q}}^{\left(0\right)}=\sqrt{Z_{1}^{\left(0\right)}Z_{2}^{\left(0\right)}}$ ) whereas the wave speed $c_{\text{Q}}$ ( $c_{\text{Q}}=4df_{\text{t}}$ ) meeting the requirement of a QAR layer is proportional to thickness *d*. If we choose $d>c_{1}/4f_{\text{t}}$ , the wave speed $c_{\text{Q}}$ inside the QAR layer becomes larger than $c_{1}$ . Since the continuity of p and $u_{z}$ at each interface requires the conservation of transverse momentum or the transverse wave vector, an observation known as Snell’s law, total internal reflection occurs at an incident angle $\theta_{1}$ larger than $\theta^{*}\equiv \sin^{-1}\frac{c_{1}}{c_{\text{Q}}}$ . Figure 5B shows the resulting total internal reflection for incident angles above the critical angle $\theta^{*}\equiv \sin^{-1}\left(3/4\right)$ when the thickness of the QAR layer is set as $d=c_{1}/3f_{\text{t}}$ . Above the critical angle, the reflectance becomes unity and the QAR layer blocks the wave being transmitted to the second medium, ceasing to function as an anti-reflective layer at $\theta_{1}\geq \theta^{*}$ . On the other hand, the wave speed inside the UAIM layer is given by $c_{\text{U}}=c_{1}/\sqrt{{\left(1/\gamma \right)}^{2}+\sin^{2}\theta_{1}}$ so that $\theta_{\text{U}}\equiv \sin^{-1}\left(\frac{c_{\text{U}}}{c_{1}}\sin\;\theta_{1}\right)$ , which is smaller than π/2 for all f and $\theta_{1}$ . Therefore, as shown in Figure 6C, one can design a constant UAIM layer without total internal reflection unless $c_{2}>c_{1}$ .

### 3.3 Realistic design of UAIM layer and its anti-reflective performance

Material parameters for UAIM introduced in (2.2.8) are nonlocal and divergent in the limit of vanishing layer thickness and 90° glancing. Practically, if we avoid these extreme cases and make a moderate restriction of frequency band and angle range, we can design an approximate universal acoustic impedance matching (aUAIM) layer utilizing metamaterials. Figure 6 shows a schematic design of aUAIM layer and its performance in anti-reflection. The double-layer structure, with two layers sandwiching a subwavelength gap layer of different material, can possess spatial dispersion due to multiple reflection and structural inhomogeneity. The induced dispersion can meet the requirement of UAIM spatio-temporal dispersion approximately within the limited spectral and angular ranges. As shown in Figure 6A, the top and the bottom layer are composed of same material, having mass density $\rho_{\text{L}}=5377\;{\text{kg/m}}^{-3}$ and bulk modulus $K_{\text{L}}=28.3\;\text{GPa}$ , and the gap layer is made of a material with $\rho_{\text{G}}=3770\;{\text{kg/m}}^{-3}$ and $K_{\text{G}}=3.27\;\text{GPa}$ . In practice, these parameter values can be achieved with a composite metamaterial through the homogenization of matrix-inclusion composite scheme [26]. By controlling the filling ratio and the shape of inclusion materials, effective mass densities and bulk moduli for each layer can be specifically designed.

An ideal QAR layer and the aUAIM layer of the same thickness d=359 μm are compared through numerical calculation using the Acoustics module in COMSOL Multiphysics. They are all in excellent agreement with analytic calculations of reflectance and transmittance following the transfer matrix method (Figure 6B and C). Here, a QAR layer with a specific acoustic impedance of $Z_{\text{Q}}$ = 6.71 MRayl is designed for a target frequency of $f_{\text{Q}}=1.7\;\text{MHz}$ . The aUAIM layer of the same total thickness possessing the dispersions described in Eq. (2.2.8), is used in the numerical calculations. Without matching layers, 82% of the acoustic wave reflection occurs at the interface between the piezoelectric material and the water for a normal incidence, and the reflectance increases as the incident angle increases (Figure 6D). Even though the QAR layer may successfully remove reflection for a normal incidence, the −10 dB bandwidth is about 17% (Figure 6E), whereas the aUAIM exhibits −10 dB bandwidth of 66% (Figure 6F) as well as covering much wider angular range.

## 4 Conclusions

In this work, we established a theory of universal acoustic impedance matching and provide an example of a constant UAIM layer that enables the perfect transmission of acoustic waves independent of frequency and incident angle. The spectral and angular dispersion properties of the constant UAIM layer were clarified and the absence of total internal reflection in the UAIM layer was explained. Moreover, we suggested the practical design of acoustic metamaterials enabling UAIM within the finite range of frequency and incident angle, and demonstrated its performance numerically. We emphasize that a practical realization of the UAIM layer still needs to be investigated and that possible solutions can be found by exploiting the exotic dispersion of non-local acoustic metamaterials. Finding a design rule for metamaterials realizing on-demand spatio-temporal dispersion is an important obstacle to overcome. We believe that our work presents a new direction for research into acoustic impedance matching and acoustic metamaterials.
